# Synergetic Contributions of High Quenching Concentration and Tuned Square Antiprism Geometry Boosting Far‐Red Emission of Eu^3+^ with Near‐Unit Efficiency

**DOI:** 10.1002/advs.202415989

**Published:** 2025-01-10

**Authors:** Hong Li, Asif Ali Haider, Zhi Xie, Conglin Liu, Hongzhi Zhang, Hongming Jiang, Junpeng Li, Jing Zhu

**Affiliations:** ^1^ Yunnan Key Laboratory of Electromagnetic Materials and Devices National Center for International Research on Photoelectric and Energy Materials School of Materials and Energy Yunnan University Kunming 650091 China; ^2^ College of Mechanical and Electrical Engineering Fujian Agriculture and Forestry University Fuzhou Fuzhou 350002 China

**Keywords:** Eu3+, Far‐red phosphor, plant growth lighting, Quenching concentration, Square antiprism

## Abstract

Far‐red phosphors have emerged as a desirable research hotspot owing to their critical role in promoting plant growth. Especially, Eu^3+^ ions typically present the ^5^D_0_→^7^F_J_ (J = 0, 1, 2, 3, 4) transitions, which overlap with the far‐red light required for plant photosynthesis. However, achieving high‐efficiency far‐red emission of Eu^3+^ remains challenging due to weak ^5^D_0_→^7^F_4_ transition and concentration quenching. The study constructs two anomalously efficient far‐red garnet phosphors A_3_Sc_2_C_3_O_12_ (A = Y^3+^, Gd^3+^. C = Al^3+^, Ga^3+^):Eu^3+^. A high‐resolution STEM measurement equipped with an aberration corrector provides the direct proofs for both the [EuO_8_] configuration‐dependent strong ^5^D_0_→^7^F_4_ and the origin of high quenching concentration. Excitedly, a two‐component substitution (replacing Y^3+^‐Al^3+^ with Gd^3+^‐Ga^3+^) triggers a near‐unity internal quantum efficiency (IQE = 99.01%) and high external quantum efficiency (EQE = 38.73%) in Gd_3_Sc_2_Ga_3_O_12_:60%Eu^3+^, resulting from the effective modulation of ^5^D_0_→^7^F_4_/^7^F_2_ transitions. A far‐red LEDs device based on Gd_3_Sc_2_Ga_3_O_12_:60%Eu^3+^ exhibits an output power of 113 mW at 300 mA. Subsequently, practical applications for promoting plant growth underscore the significance of these findings. This work opens a new path for the development of highly efficient far‐red phosphors via the synergistic effect of Eu^3+^ square antiprism configuration and high quenching concentration.

## Graphical abstract

Two isostructural garnet phosphors A_3_Sc_2_C_3_O_12_ (A = Y^3+^, Gd^3+^. C = Al^3+^, Ga^3+^):Eu^3+^ are developed to deal with the puzzles of weak ^5^D_0_→^7^F_4_ transition and concentration quenching. Advanced STEM technology provides the direct proofs for both the [EuO_8_] configuration‐dependent strong ^5^D_0_→^7^F_4_ and the origin of high quenching concentration. Meanwhile, the ^5^D_0_→^7^F_4_/^7^F_2_ transition is effectively modulated to realize a near‐unity IQE.

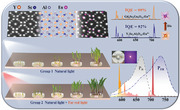

## 1 Introduction

Light is essential throughout the entire plant growth cycle. Plant pigments (chlorophyll a, chlorophyll b, phytochrome red (P_R_), and far‐red‐absorbing phytochrome (P_FR_)) absorb light for photosynthesis.^[^ ^1^, ^2^, ^3^ ^]^ P_R_ and P_FR_ primarily absorb red and far‐red light to regulate flowering and growth of leaves and stems.^[^ ^4^, ^5^, ^6^ ^]^ Far‐red phosphor‐converted light‐emitting diodes (pc‐LEDs) are considered ideal artificial light sources for plant growth due to their compact size, eco‐friendliness, and long lifespan.^[^ ^7^, ^8^, ^9^, ^10^ ^]^ As a result, the development of highly efficient far‐red phosphors has become a research hotspot.

Currently, available far‐red phosphors are mostly activated by Mn^4+^, Eu^2+^, or Cr^3+^.^[^ ^11^, ^12^, ^13^ ^]^ Meanwhile, these materials encounter the troubles of complicated synthesis and low luminescence thermostability, hindering practical plant lighting applications. Generally, Eu^3+^, as a widely used trivalent rare earth ion, presents a series of red/far‐red emissions in the range of 570–750 nm under UV light excitation.^[^ ^14^, ^15^, ^16^, ^17^ ^]^ For the adjustment and improvement of Eu^3+^ luminescence, the diverse strategies can be employed, such as delaying concentration quenching, co‐doping sensitizer and activator, reducing local symmetry.^[^ ^18^, ^19^, ^20^, ^21^ ^]^ A lot of Eu^3+^‐activated phosphors with high color purity and stable physicochemical properties were reported. However, most of them present dominant ^5^D_0_→^7^F_2_/^7^F_1_ (orange–red/red light) transitions, such as Cs_3_GdGe_3_O_9_:Eu^3+^, Na_2_Y_2_TeO_4_(BO_3_)_2_:Eu^3+^, and Na_2.5_Zr_2_Si_1.5_P_1.5_O_12_:Eu^3+^.^[^ ^22^, ^23^, ^24^ ^]^ Although Eu^3+^‐activated far‐red phosphors with strong ^5^D_0_→^7^F_4_ transition have been developed in the past two years, the relatively low Eu^3+^ doping concentration results in poor external quantum efficiency (EQE).^[^ ^4^ ^]^ Elevating the Eu^3+^ concentration often leads to an increased non‐radiative energy transfer, producing concentration quenching due to a short Eu^3+^−Eu^3+^ distance. A large distance between Eu^3+^ ions is crucial for achieving high luminous efficiency. Recently, high doping concentration of Eu^3+^ was achieved in some Eu^3+^‐activated red (^5^D_0_→^7^F_2_) and far red (^5^D_0_→^7^F_4_) phosphors reported successfully. For instance, in Cs_3_GdGe_3_O_9_, Eu^3+^ can fully substitute Gd^3+^ with dominant ^5^D_0_→^7^F_2_ transition. The Eu^3+^−Eu^3+^ distance was as large as 6.836 Å. Such large distance effectively suppressed non‐radiative energy transfer, leading to efficient red emission.^[^ ^22^ ^]^ Similarly, the inter‐ and intralayer distances of Ba_6_Gd_2_Ti_4_O_17_:90%Eu^3+^ restricted the direction of energy migration, successfully avoiding concentration quenching.^[^ ^25^ ^]^ Another report was that the doping concentration of Eu^3+^ in Ca_3_Al_2_Ge_3_O_12_ with strong ^5^D_0_→^7^F_4_ transition is 40%.^[^ ^26^ ^]^ A far‐red LEDs based on Ca_3_Al_2_Ge_3_O_12_:40%Eu^3+^ has an output power of 27.3 mW at 200 mA, showing potential lighting applications in plant growth. The previous studies have inspired us to investigate the synergetic contributions of strong ^5^D_0_→^7^F_4_ transition and high doping concentration for developing highly efficient Eu^3+^‐activated far‐red phosphors.

Garnet‐structure materials with the general formula of A_3_B_2_C_3_O_12_ are promising phosphor hosts due to their rich cation sites and rigid structure. The A, B, and C sites correspond to dodecahedron [AO_8_] (square antiprism), octahedron [BO_6_], and tetrahedron [CO_4_], respectively.^[^ ^27^, ^28^ ^]^ These polyhedra are interconnected to construct a stable 3D framework structure. The elements Y, Lu, Mg, Ca, Sr, Gd, La, and Na can occupy the A site. The B site can accommodate the Mg, Hf, Sc, Y, Zr, Ga, Sn, and Al elements. The elements Al, Ga, Si, Ge, and Ti can enter the C site. Consequently, there are structural and compositional tunability for the material family. Notably, based on previous reports from domestic and foreign counterparts, the [AO_8_] square antiprism configuration is conducive to the ^5^D_0_→^7^F_4_ transition of Eu^3+^.^[^ ^29^ ^]^ Obviously, garnet‐structured materials are ideal hosts for achieving efficient far‐red emission of Eu^3+^. Nevertheless, the rich cation sites in the structure bring the trouble for precisely determining emitting centers. For example, Y_2_Mg_2_Al_2_Si_2_O_12_:Eu^3+^ phosphor with excellent luminescence thermostability was found.^[^ ^30^ ^]^ Unfortunately, it remained unclear for the Eu^3+^ substitution due to the especial A sites occupied simultaneously by Y^3+^ and Mg^2+^. Similarly, in Ca_2_LaHf_2_Al_3_O_12_:Eu^3+^, there are Ca^2+^ and La^3+^ ions in the A sites, which Eu^3+^ can enter.^[^ ^31^ ^]^ The previous studies relied heavily on theoretical calculations and spectral analysis to determine the site occupancy of Eu^3+^.

In this work, two far‐red garnet phosphors A_3_Sc_2_C_3_O_12_ (A = Y^3+^, Gd^3+^. C = Al^3+^, Ga^3+^):Eu^3+^ with high internal quantum efficiencies (IQE > 90%) are reported. Both phosphors exhibit strong ^5^D_0_→^7^F_4_ transitions. The garnet structure is thoroughly investigated via atomic resolution scanning transmission electron microscopy with high‐angle annular dark‐field (STEM‐HAADF), directly confirming that the Eu^3+^ occupancy with square antiprismatic configuration. The result accounts for strong ^5^D_0_→^7^F_4_ transition and high quenching concentration (60 mol%). Meanwhile, the near‐unity IQE in Gd_3_Sc_2_Ga_3_O_12_:60%Eu^3+^ is achieved via the replacement of Gd^3+^‐Ga^3+^ for Y^3+^‐Al^3+^. Finally, an 8‐day plant growth lighting experiment is conducted using corn seeds as the test subject.

## 2 Results and Discussion

### 2.1 [EuO_8_] Square Antiprism Configuration

**Figure 1:**
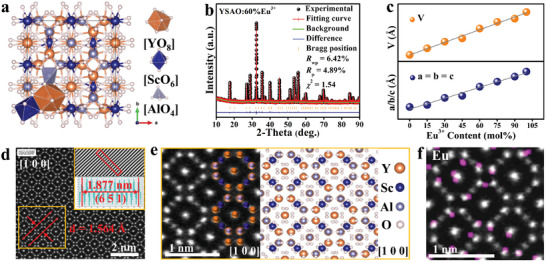
a) Crystal structure of YSAO. b) Rietveld refinement pattern of YSAO:60%Eu^3+^. c) Changes in the cell parameters (*a*/*b*/*c* and *V*) with Eu^3+^ content. d) STEM–HAADF image. e) Magnified atomic arrangement and corresponding structure along the zone axis [100]. f) Atomic‐scale EDS mapping of Eu^3+^.

**Figure** **1**a shows the crystal structure of Y_3_Sc_2_Al_3_O_12_ (YSAO) along *c*‐axis, which belongs to cubic garnet structure with *Ia* $\bar{3}$ *d* (230) space group. The structure contains [YO_8_] square antiprism, [ScO_6_] octahedra, and [AlO_4_] tetrahedra. According to the replacing principle of similar ionic radii and equal valence state, Eu^3+^ is likely to occupy Y^3+^ and Sc^3+^ sites. Subsequently, the formation energy of Eu^3+^ substitution in YSAO was calculated using the following Equation (1):^[^ ^32^, ^33^ ^]^

(1) $$E_{\mathrm{form}}=E_{\mathrm{doped}}\;-\;E_{\mathrm{pure}}\;-\;n\;\times \;E_{\mathrm{dopant}}+m\;\times \;E_{\mathrm{atom}}$$

where *E*_doped_ and *E*_pure_ represent the total energies of the host with or without dopant, separately. *E*_dopant_ and *E*_atom_ denote the atomic energies of the dopant and the atom replaced by dopant, separately. n and m denote the number of the dopant and the atom replaced by dopant, separately. The formation energy of Eu^3+^ at the Y^3+^ site is significantly lower than that at the Sc^3+^ site (Figure S1, Supporting Information), indicating that Eu^3+^ is more likely to occupy the Y^3+^ site. X‐ray diffraction (XRD) and the refined XRD results confirm the acceptable phase purity (Figure S2; Tables S1–S3, Supporting Information). A representative refined XRD pattern of YSAO:60%Eu^3+^ is shown in Figure 1b. The lattice parameters and volume increase linearly with increasing Eu^3+^ concentration, as shown in Figure 1c. The ionic radius of Eu^3+^ is larger than that of Y^3+^, indicating that Eu^3+^ is successfully populated into the Y^3+^ site.

To further understand the arrangement of all ions and the specific occupation sites of Eu^3+^, high‐resolution scanning transmission electron microscopy (STEM) coupled with high‐angle annular dark‐field (HAADF) imaging was performed on YSAO:60%Eu^3+^. Figure 1d presents a clear STEM‐HAADF image along the [100] zone axis, where the bright spots represent the cation positions. Due to the low scattering intensity, the oxygen ions are barely visible. The cations exhibit a highly regular arrangement, indicating excellent crystallinity. The calculated interplanar spacing is 1.564 Å, corresponding to the (651) crystal plane. Figure 1e shows the STEM‐HAADF image at the 1 nm scale, displaying an ideal garnet structure without any defects. The investigation perfectly illustarates the crystal structure of YSAO along the [100] zone axis, which is supported by the electron diffraction patterns along the [100] zone axis (Figure S3, Supporting Information). The bright spots represent Y^3+^ ions (yellow). The relatively dimmer spots correspond to Sc^3+^ ions (blue). Additionally, the Al^3+^ ions (light‐purple) are clearly observed.

Figure 1f shows the atomic‐scale energy dispersive spectrometer (EDS) mapping image of Eu^3+^, providing a clear substitution observation by the combined analysis of those for Y^3+^, Sc^3+^, and Al^3+^ (Figure S4, Supporting Information). The purple spots represent the positions occupied by Eu^3+^, revealing that Eu^3+^ exclusively replace Y^3+^ rather than Sc^3+^, which is consistent with the formation energy calculation results. The powder sample consists of irregular small particles with an approximate diameter of 4.73 µm (Figure S5, Supporting Information).

### 2.2 High Quenching Concentration of Eu^3+^

**Figure 2:**
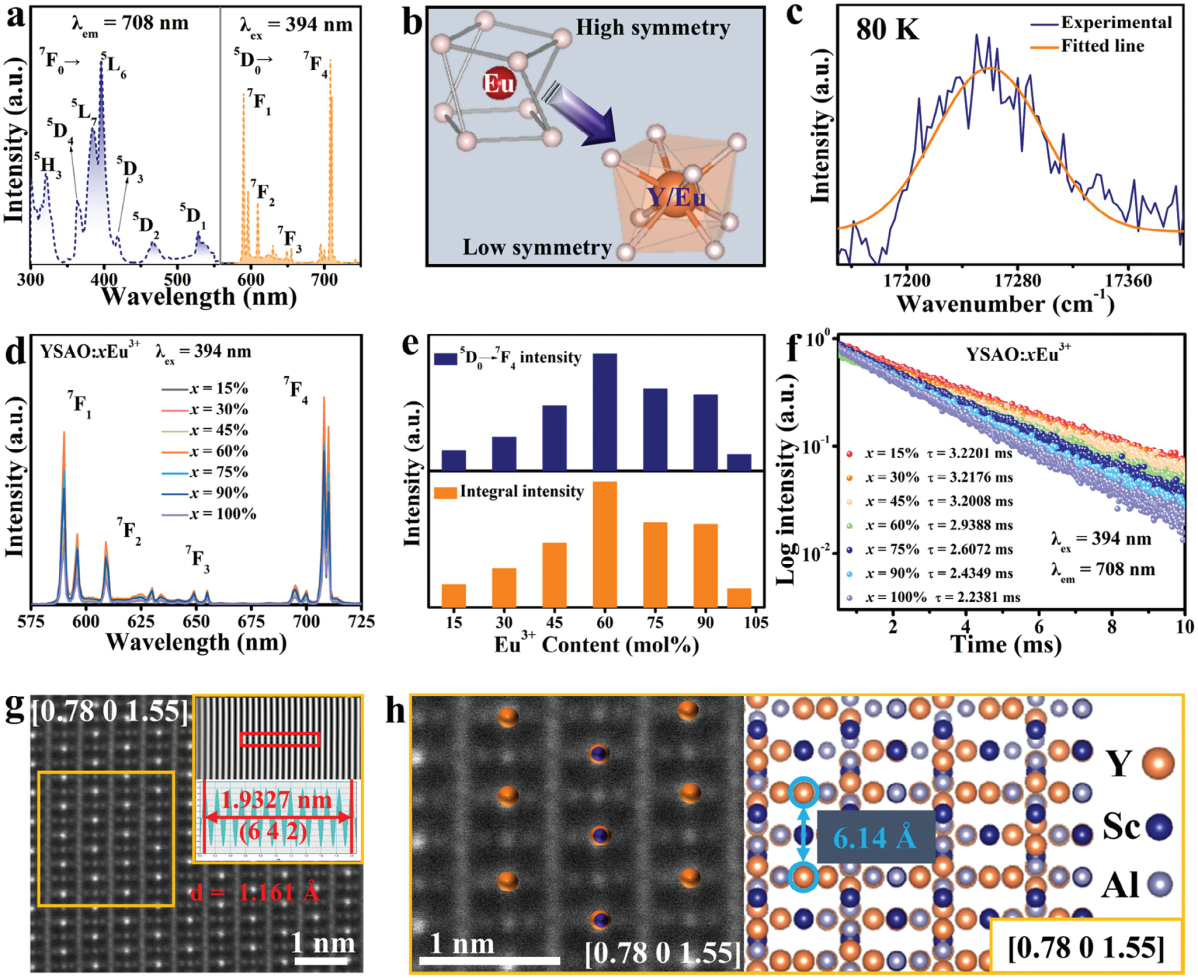
a) PLE and PL spectra of YSAO:60%Eu^3+^. b) Ideal [EuO_8_] and distorted [Y/EuO_8_] square antiprism configuration. c) Gaussian fitting of ^7^F_0_→^5^D_0_ transition at 80 K. d) PL spectra of YSAO:*x*Eu^3+^. e) Eu^3+^ content‐dependent ^5^D_0_→^7^F_4_ intensity and integral intensity. f) Decay curves. g) STEM–HAADF image. h) Magnified atomic arrangement and corresponding structure along the zone axis [0.78 0 1.55].

**Figure** **2**a shows the photoluminescence excitation (PLE) and photoluminescence (PL) spectra of YSAO:60%Eu^3+^ at room temperature. A series of sharp PLE peaks arising from the characteristic 4f‐4f transitions of Eu^3+^ are observed. Specifically, the PLE peaks at 319, 362, 382, 394, 417, 465, and 527 nm correspond to the ^7^F_0_→(^5^H_3_, ^5^D_4_, ^5^L_7_, ^5^L_6_, ^5^D_3_, ^5^D_2_, and ^5^D_1_) transitions of Eu^3+^, respectively.^[^ ^34^, ^35^ ^]^ The strongest PLE peak at 394 nm aligns well with the NUV chip (395 nm). Under 394 nm excitation, PL peaks at 590, 609, 649, and 708 nm correspond to the ^5^D_0_→^7^F_1_, ^5^D_0_→^7^F_2_, ^5^D_0_→^7^F_3_, and ^5^D_0_→^7^F_4_ transitions of Eu^3+^, respectively. The electric dipole ^5^D_0_→^7^F_2_ transition is significantly weaker than the magnetic dipole ^5^D_0_→^7^F_1_ transition, indicating that Eu^3+^ occupies a highly symmetric environment in YSAO.^[^ ^36^, ^37^ ^]^ Notably, ^5^D_0_→^7^F_4_ is the dominant transition, which differs from the typical luminous properties of Eu^3+^. Typically, *D*_4d_ symmetry favors the ^5^D_0_→^7^F_4_ transition.^[^ ^38^, ^39^ ^]^ As shown in Figure 2b, an undistorted square antiprism exhibits *D*_4d_ symmetry, where the ^5^D_0_→^7^F_2_ transition is forbidden, while the ^5^D_0_→^7^F_4_ transition is allowed.^[^ ^40^ ^]^ In a slightly distorted square antiprism, local symmetry is reduced, resulting in the presence of the ^5^D_0_→^7^F_2_ transition. In YSAO:60%Eu^3+^, the [Y/EuO_8_] polyhedron resembles a slightly distorted square antiprism, exhibiting a strong ^5^D_0_→^7^F_4_ and weak ^5^D_0_→^7^F_2_ transitions. The ^7^F_0_→^5^D_0_ transition can be used to determine the number of Eu^3+^ occupied sites.^[^ ^40^ ^]^ Therefore, PLE spectra (λ_em_ = 590, 608, and 708 nm) for the ^7^F_0_→^5^D_0_ transition at 80 K were tested (Figure S6, Supporting Information), presenting only one peak. Gaussian fitting for the strongest peak (λ_em_ = 708 nm) is displayed in Figure 2c, indicating the existence of only one independent emission center. This result is consistent with the formation energy calculations and STEM‐HAADF observations, further confirming that Eu^3+^ exclusively occupies the Y^3+^ sites. Figure 2d shows the PL spectra of YSAO:*x*Eu^3+^. All spectra exhibit consistent shapes except for intensity. As shown in Figure 2e, the ^5^D_0_→^7^F_4_ intensity and integrated intensity increase with the increase of Eu^3+^ concentration, peaking at *x* = 60% before declining due to enhanced non‐radiative energy transfer. Concentration quenching is caused by electric dipole‐dipole interactions. The corresponding analysis and calculations are provided in the Supporting Information (Figure S7, Supporting Information). The decay curves of YSAO:*x*Eu^3+^ are shown in Figure 2f. All the curves are well‐fitted by a single exponential function. As *x* increases, the decay time decreases from 3.2201 to 2.2381 ms due to increased non‐radiative transitions between Eu^3+^.

Significantly, the quenching concentration of Eu^3+^ reaches as high as 60 mol%. The theoretical quenching concentration is calculated to be 61.37% (Figure S8, Supporting Information), which corroborates the experimental value (detailed analysis and calculations are discussed in the Supporting Information). Such a high quenching concentration is linked to the structure of the YSAO. Figure 2g shows the STEM‐HAADF image along the zone axis [0.78 0 1.55], with the selected region corresponding to the (6 4 2) crystal plane. The corresponding electron diffraction patterns are shown in Figure S9 (Supporting Information). Magnified atomic arrangement and crystal structure images are presented in Figure 2h. The atomic arrangement appears as a layered structure, with a distance of 6.14 Å between the Y atoms in the adjacent layers. Since Eu^3+^ occupies only the Y^3+^ sites, the large distance between square antiprisms [Y/EuO_8_] effectively inhibits non‐radiative energy transfer, achieving high quenching concentration.

### 2.3 Two‐Component Substitution of Gd^3+^‐Ga^3+^ for Y^3+^‐Al^3+^

To explore the influence of local field on Eu^3+^ luminescence, a two‐component substitution (replacing Y^3+^‐Al^3+^ with Gd^3+^‐Ga^3+^) was employed to synthesize Gd_3_Sc_2_Ga_3_O_12_ (GSGO) and GSGO:60%Eu^3+^ samples. The XRD results indicate that both samples have the same structure as YSAO (Figure S10a—c; Tables S1–S3, Supporting Information). The EDS results (Figure S10d,e, Supporting Information) further indicate that GSGO and GSGO:60%Eu^3+^ are successfully synthesized. The stability of the garnet structure is evaluated using the garnet tolerance factor (*τ*) as follows:^[^ ^41^, ^42^ ^]^

(2) $$\tau =\frac{3\sqrt{{\left(R_{B}+R_{O}\right)}^{2}-\frac{9}{4}{\left(R_{A}+R_{O}\right)}^{2}}}{2\left(R_{C}+R_{O}\right)}$$

where *R*_A_, *R*_B_, and *R*_C_ represent the average ion radii at positions A, B, and C respectively. *R*_O_ is the ionic radius of O^2−^. When *τ* approaches 1, the structure becomes more stable. If the *τ* value deviates significantly from 1, the structure is unstable.^[^ ^43^ ^]^ The τ values for YSAO and GSGO are calculated to be 1.096 and 1.112, respectively, indicating that YSAO is more stable than GSGO. The calculated formation energy of Eu^3+^ substitution in GSGO is shown in Figure S11 (Supporting Information), indicating that Eu^3+^ exclusively occupies Gd^3+^ sites.

**Figure 3:**
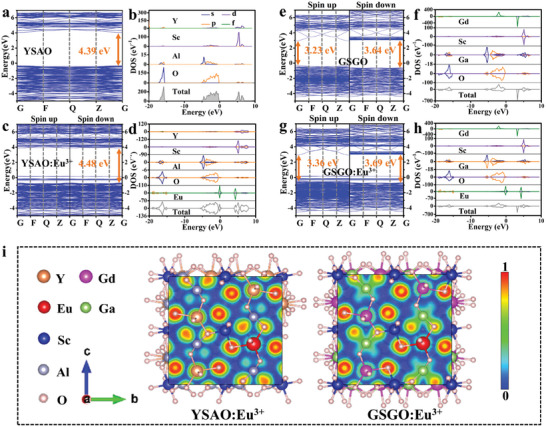
a,c) Energy bands of YSAO and YSAO:Eu^3+^. b,d) Corresponding total and partial DOS. e,g) Energy bands of GSGO and GSGO:Eu^3+^. f,h) Corresponding total and partial DOS. i) ELF topological comparative study for YSAO:Eu^3+^ and GSGO:Eu^3+^.

The luminescent properties of a material are intrinsically linked to its electronic structure. Hence, the band structures of both the host and Eu^3+^‐doped samples were calculated using density functional theory (DFT). As shown in **Figure** **3**a, the YSAO possesses a direct bandgap with the value of 4.39 eV. Figure 3b displays the corresponding density of states (DOS), indicating that the valence band maximum (VBM) is primarily composed of O‐2p states, and the conduction band minimum (CBM) is mainly derived from the Sc‐3d and Y‐4d states. Therefore, light absorption of YSAO is mainly associated with electron transitions from O^2−^ to Sc^3+^ and Y^3+^. To explore the impact of Eu^3+^ doping on the electronic structure of YSAO, 5 of 24 Y atoms were replaced by Eu atoms in the unit cell of YSAO, creating the Eu doing concentration of 20.8%, which is near the experimental doping concentration of 20% (Y_2.4_Sc_2_Al_3_O_12_:0.6Eu^3+^). Figure 3c presents the band structure of YSAO:60%Eu^3+^. Due to different characteristics, the band structures and DOS for both spin‐up and spin‐down states are displayed. It is shown that some impurity energy levels are presented between the valence and conduction bands of YSAO host after the Eu^3+^ doping, and they are derived from Eu‐4f states by the analysis of DOS in Figure 3d. It should be noted that the other characteristics are consistent with the host: the VBM mostly comes from the O‐2p states, while the CBM is dominated by the Sc‐3d and Y‐4d states. But the introduction of Eu‐4f impurity bands leads to an enlarged bandgap of YSAO:60%Eu^3+^ to 4.48 eV, because the energy of the valence band at the G point changed after the Eu doping. Figure 3e illustrates the band structure of GSGO, which shows direct bandgap with a value of 3.23 eV for the spin‐up state and 3.64 eV for the spin‐down state. According to the DOS (Figure 3f), the VBM is mainly composed of O‐2p states, while the CBM is mainly derived from Gd 4f states. Figure 3g presents the band structure of GSGO:60%Eu^3+^, where the bandgap is enlarged compared to GSGO. Similarly, the Eu^3+^ impurity energy levels appear above the VBM of GSGO host, as shown in Figure 3h. The UV–vis diffuse reflectance (DR) spectra of YSAO:60%Eu^3+^ and GSGO:60%Eu^3+^ are shown in Figure S12 (Supporting Information). Both exhibit strong broadband absorption in the 200–300 nm range. Furthermore, a series of characteristic Eu^3+^ absorption peaks in the 300–600 nm range are observed. Based on the DR data, the Kubelka‐Munk transformation is applied to determine the optical bandgap as follows:^[^ ^44^, ^45^ ^]^

(3) $$F\;\left(R\right)={\left(1-R\right)}^{2}\;/2R=K/S$$

(4) $${\left[F\left(R\right)hv\right]}^{1/n}=A\left(hv-\;E_{g}\right)$$

where *R*, *hv*, and *A* are the reflectance, photon energy, and absorption constant, respectively. The n values of 1/2 and 2 correspond to the direct and indirect allowed transitions in the same order. The electronic transitions of YSAO:60%Eu^3+^ and GSGO:60%Eu^3+^ belong to direct allowed transitions (n = 1/2). The transformed images are shown in the insets, where the intersection of the two fitting lines represents the bandgap values. The bandgap values for YSAO:60%Eu^3+^ and GSGO:60%Eu^3+^ are 4.09 and 3.85 eV, respectively. The experimental bandgap values are basically in accordance with the theoretical values except for a little difference owing to the inherent limitation of DFT method.^[^ ^46^ ^]^

To evaluate the charge transfer process originating from Eu to O atoms in YSAO:Eu^3+^ and GSGO:Eu^3+^, the Electron Localization Function (ELF) analysis was performed. As shown in Figure 3i, the isosurfaces are primarily concentrated around atoms rather than being uniformly distributed between Eu and O atoms, indicating typical ionic bonding in the Eu−O bonds. The electron cloud color represents the distribution of electrons around the atoms: green indicates the electrons provided by the Eu atoms, and red indicates the electrons accepted by the O atoms. The degree of redness of the electron cloud reflects the degree of electron localization on the O atom. In GSGO:60%Eu^3+^, the red electron cloud around the O atoms is lighter than that in YSAO:60%Eu^3+^, suggesting that electron localization on the O atoms is weaker in GSAO:60%Eu^3+^. That is, the Eu─O bond in GSAO:60%Eu^3+^ has lower degree of ionicity.

**Figure 4:**
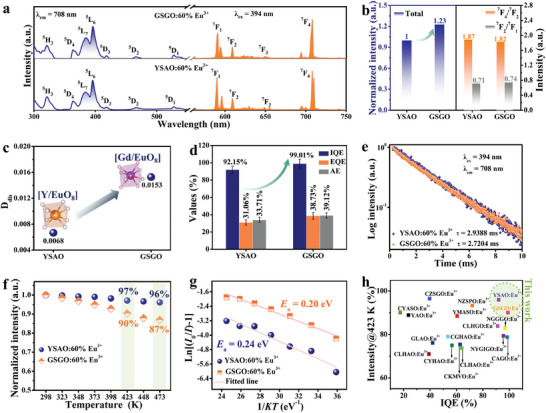
Comparison of the luminous properties of YSAO:60%Eu^3+^ and GSGO:60%Eu^3+^. a) PLE and PL spectra. b) Total integral intensity, ^7^F_4_/^7^F_2_ ratio, and ^7^F_2_/^7^F_1_ ratio. c) Distortion of the [Y/EuO_8_] and [Gd/EuO_8_] polyhedra. d) QE result. e) Decay curves. f) Normalized emission integrated intensities as a function of temperature. g) Fitting diagrams of *E*_a_. h) Comparison of IQE and thermostability.

**Figure** **4**a presents the PLE (λ_em_ = 708 nm) and PL (λ_ex_ = 394 nm) spectra of YSAO:60%Eu^3+^ and GSGO:60%Eu^3+^. Both samples exhibit the same PLE/PL shapes and locations except for intensity, resulting from the 4f‐4f transitions of Eu^3+^. The charge transfer band (CTB) positions in the two samples are different (Figure S13, Supporting Information). The CTB of YSAO:60%Eu^3+^ is centered at 238 nm, whereas GSGO:60%Eu^3+^ shows a CTB at 255 nm. CTB is influenced by the covalency of the Eu−O bond and the coordination environment of Eu^3+^,^[^ ^47^, ^48^ ^]^ and its position can be calculated using Jørgensen's Equation (5):^[^ ^49^ ^]^

(5) $$\sigma =\left[\chi_{\mathrm{opt}}\left(X\right)-\chi_{\mathrm{uncorr}}\left(M\right)\right]30\;\times \;10^{3}\;cm^{-1}$$

where σ represents the energy of the CTB, χ_opt_(X) denotes the optical electronegativity of ligand ion, and χ_uncorr_(M) denotes the optical electronegativity of central cation. Based on the spectral data and Equation (5), the differences in the electronegativity values [χ(O^2−^) − χ(Eu^3+^)] for YSAO:60%Eu^3+^ and GSGO:60%Eu^3+^ are calculated to be 1.394 and 1.305, respectively. The decrease in the electronegativity difference indicates the increased covalency of the Eu−O bond, which is consistent with the result of the ELF analysis. The stronger covalency of the Eu−O bond in GSGO:60%Eu^3+^ leads to a lower CTB energy. The PLE spectra at 80 K show that Eu^3+^ occupies only one site in the GSGO (Figure S14, Supporting Information). The comparisons of the total intensity and the intensity ratio of different transitions are shown in Figure 4b. The total integrated emission intensity of GSGO:60%Eu^3+^ is 1.23 times that of YSAO:60%Eu^3+^. The ^7^F_4_/^7^F_2_ ratio decreased, whereas the ^7^F_2_/^7^F_1_ ratio slightly increased. The changes in the ^5^D_0_→^7^F_2_ transitions are related to the local structural symmetry variations of the [LnO_8_] polyhedron. Typically, the distortion index (*D_dis_*) of a polyhedron is used to reflect the degree of the local structural symmetry. A larger *D_dis_* indicates lower structural symmetry. Based on the structural refinement results, the *D_dis_* of the [LnO_8_] polyhedron is calculated using the following Equation (6):^[^ ^50^ ^]^

(6) $$D_{dis}=\frac{1}{n}\sum_{i=1}^{n}\frac{\left|l_{i}-l_{av}\right|}{l_{av}}$$

where *l*_i_ is the distance of Ln─O bond and *l*_av_ is average bond length. As shown in Figure 4c, the *D*_dis_ values of [Y/EuO_8_] and [Gd/EuO_8_] are 0.0068 and 0.0153, respectively, disclosing that the reduced local structural symmetry contributes to the enhancement of the ^5^D_0_→^7^F_2_ transition after the double substitution of Gd^3+^‐Ga^3+^ for Y^3+^‐Al^3+^. Combined with LnTeBO_5_ (Ln = La^3+^, Y^3+^,Gd^3+^):Eu^3+^ and K_6_Bi_13_(PO_4_)_15_:Eu^3+^ reported by our group,^[^ ^38^, ^39^ ^]^ we preliminarily consider that the proper distortion of square antiprism for Eu^3+^ is in favour of obtaining intense ^5^D_0_→^7^F_2_/^7^F_4_ transitions. Meanwhile, the *D*_dis_ is related to the electronegativity of the central atom. A central atom with high electronegativity can cause the coordination field to deviate from ideal symmetry. Althrough the electronegativities of Y and Gd are both 1.2, the electronegativities of Al and Ga are 1.6 and 1.8, respectively, indicating that the substitution of Ga for Al induces a large tetrahedron distortion. Owing to the connection of tetrahedron and dodecahedron via sharing edge, the distortion of tetrahedron indirectly leads to the [LnO_8_] distortion. Hence, the *D*_dis_ of [Gd/EuO_8_] is higher than that of [Y/EuO_8_]. Figure 4d shows a comparison of the quantum efficiency (QE) values of the two samples. The corresponding spectra are shown in Figure S15 (Supporting Information). YSAO:60%Eu^3+^ exhibits excellent QE value. In particular, based on the synergistic effect of the distorted square antiprism configuration and high quenching concentration,^[^ ^51^ ^]^ the two‐component substitution of Gd^3+^‐Ga^3+^ for Y^3+^‐Al^3+^ effectively enhances the luminescence efficiency. GSGO:60%Eu^3+^ achieves a near‐unity internal quantum efficiency (IQE), along with an ultrahigh external quantum efficiency (EQE = 38.73%) and absorption efficiency (AE = 39.12%), surpassing most Eu^3+^‐activated garnet phosphors (Table S4, Supporting Information). As shown in Figure 4e, the decay curves conform well to a single exponential function, indicating the presence of only one luminescent center. The decay time is slightly shorter than that of YSAO:60%Eu^3+^.

To evaluate luminescence thermal stability, the PL spectra of YSAO:60%Eu^3+^ and GSGO:60%Eu^3+^ were recorded at various temperatures (Figure S16, Supporting Information). As shown in Figure 4f, both phosphors exhibit a slow decrease in the emission intensity with increasing temperature. The decline in GSGO:60%Eu^3+^ is slightly more pronounced than that in YSAO:60%Eu^3+^. Even so, at 423 and 473 K, GSGO:60%Eu^3+^ still maintains 90% and 87% of the initial PL intensity, respectively, surpassing most Eu^3+^‐activated garnet phosphors (Table S4, Supporting Information). The activation energy (*E*_a_) for thermal quenching is determined using the Arrhenius Equation (7):

(7) $$\ln\left[\left(I_{0}/I\left(T\right)\right)\;-\;1\right]=\ln A-\;\left(E_{a}/kT\right)$$

where *I*_0_ is the initial emission intensity at room temperature. *I*(*T*) represents the emission intensity at the testing temperature *T*. *A* is a constant. *k* is the Boltzmann constant (8.625 × 10^−5^ eV K^−1^).^[^ ^52^ ^]^ The values of *E*_a_ for YSAO:60%Eu^3+^ and GSGO:60%Eu^3+^ are calculated to be 0.24 and 0.20 eV, respectively (Figure 4g). Typically, *E*_a_ is proportional to the energy of CTB (Figure S17, Supporting Information). The earlier discussion has confirmed that the high covalency of the Eu─O bond in GSGO:60%Eu^3+^ leads to a decreased CTB energy, which is responsible for the reduced luminescence thermal stability. The luminescence thermostability also depends on the structural rigidity.^[^ ^53^ ^]^ Materials with stronger structural rigidity show better thermostability. The structural rigidity is typically evaluated using the Debye temperature (Θ_D_).^[^ ^54^ ^]^ Based on DFT calculations, the Θ_D_ values of YSAO and GSGO are calculated to be 736 K and 577 K, respectively, indicating that the substitution of Y^3+^‐Al^3+^ with Gd^3+^‐Ga^3+^ weakens the structural rigidity, which is consistent with the calculated τ values. In addition, materials with a large bandgap can effectively suppress the thermal ionization of electrons.^[^ ^55^ ^]^ According to the analysis of the electronic structure, the bandgap of YSAO:60%Eu^3+^ is larger than that of GSGO:60%Eu^3+^. Thus, the reduced energy of the CTB, weakened structural rigidity, and reduced bandgap lead to a slight decrease in the luminescent thermostability of GSGO:60%Eu^3+^. As depicted in Figure 4h, compared with Eu^3+^‐activated garnet phosphors reported recently, the studied phosphors are positioned in the upper right corner, indicating that YSAO:60%Eu^3+^ and GSGO:60%Eu^3+^ exhibit superior luminescence efficiency and thermostability. The detailed data information is summarized in Table S4 (Supporting Information).

### 2.4 Far‐Red Lighting of Eu^3+^ for Plant Growth

**Figure 5:**
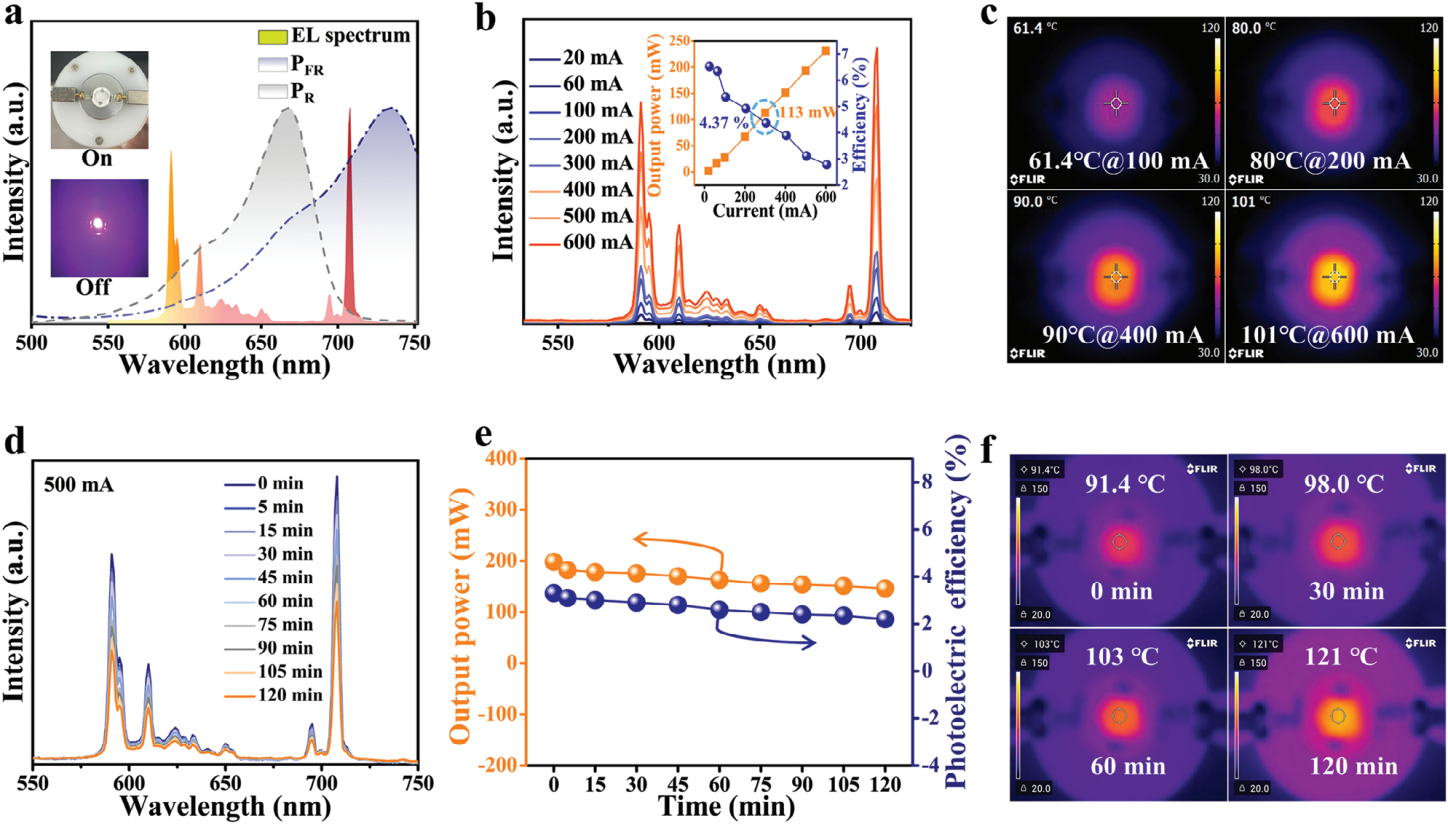
a) EL spectrum and digital photographs of far‐red LEDs at 20 mA. b) EL spectra in response to current. c) Thermal images under different current. d) EL spectra in response to running time at 500 mA. e) Time‐dependent output power and photoelectric efficiency. f) Thermal images at different times.

To demonstrate the potential application of the studied phosphors in plant lighting, a representative NUV chip (395 nm)‐excited far‐red LEDs was fabricated by combining the optimal phosphor GSGO:60%Eu^3+^ with high luminous efficiency. The EL spectrum and digital photographs of the far‐red LEDs are shown in **Figure** **5**a. At a driving current of 20 mA, the device emits bright far‐red light. Importantly, the EL spectrum partially overlaps with the absorptions of the phytochrome P_FR_ and P_R_, indicating its potential as a supplementary light source to accelerate plant growth. As shown in Figure 5b, as the driving current increases from 20 to 600 mA, the emission intensity of the device increases continuously, while all emission profiles remain consistent. The inset shows the output power and photoelectric conversion efficiency under various driven currents. Notably, at 300 mA, the device achieves an output power of up to 113 mW, surpassing most far‐red LEDs devices reported (Table S5, Supporting Information). During this process, the maximum temperature of the device only reaches 101 °C (Figure 5c), indicating that the device operates normally even under high current. To evaluate the thermal stability of the fabricated far‐red LEDs, a continuous operation test was conducted under a high current of 500 mA for 2 h. As shown in Figure 5d, all EL spectra retain consistent shapes over time except for a reduction in emission intensity. Meanwhile, the output power and optoelectronic conversion efficiency decrease slowly with the increase of operating time, as shown in Figure 5e. Notably, the device maintains high output power after 2 h, demonstrating good thermal stability. Additionally, the operating temperature gradually increases during the test, reaching 121 °C after 2 h (Figure 5f). According to the analysis of the luminescence thermostability, the GSGO:60%Eu^3+^ phosphor can maintain ≈93% of the luminescence intensity at 121 °C (Figure 4f). Therefore, the performance of GSGO:60%Eu^3+^ phosphor meets the requirements for far‐red lighting application.

**Figure 6:**
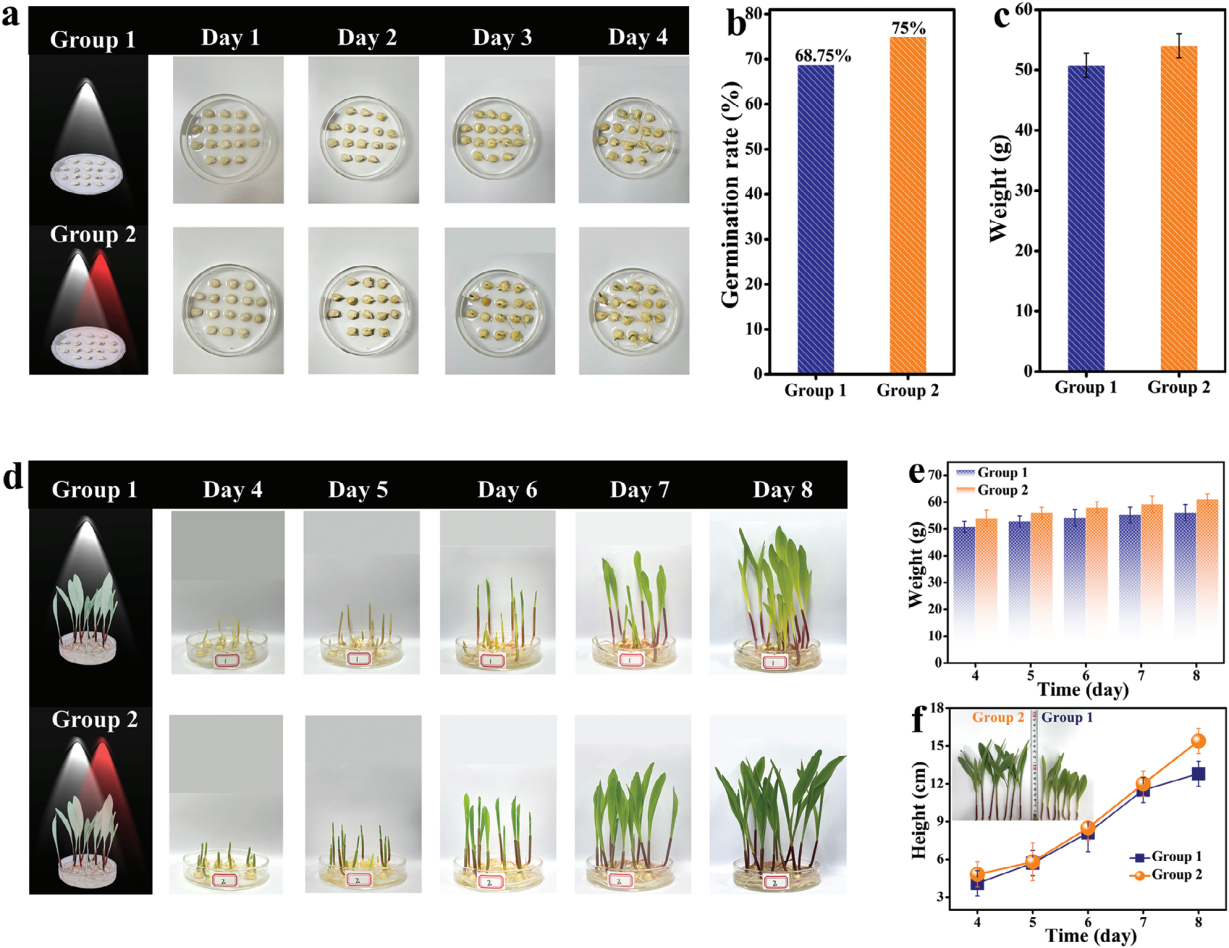
a) Growth control charts for corn seeds under natural light (Group 1) and natural light combined with supplementary far‐red light (Group 2). b) Germination rate statistics. c) Weight statistics. d) Growth control charts for corn seedlings under natural light (Group 1) and natural light combined with supplementary far‐red light (Group 2). e) Weight and f) Height statistics.

To verify the promotion effect of the prepared far‐red LEDs on plant growth, an 8‐day growth experiment was conducted using corn seeds. The seeds were evenly divided into two groups (16 seeds per group): Group 1 was placed under natural light, and Group 2 under natural light supplemented with light from the prepared far‐red LEDs. Photographs and weights were recorded every alternate day to monitor the growth status of both groups. As shown in **Figure** **6**a, there is no change in either group of seeds on the first and second days. Starting on the third day, the differences become apparent. The seeds of Group 2 begin to root and germinate, whereas the seeds of Group 1 only root without germinating. By the fourth day, the germination rate of Group 1 is 68.75%, while that of Group 2 is 75%, As depicted in Figure 6b. Additionally, the weight of Group 2 exceeds that of Group 1 (Figure 6c). Starting on the fourth day, the growth status of the two groups of corn seedlings is recorded (Figure 6d). By the fifth day, both groups begin to grow green leaves, with Group 2 showing greener leaves. By the sixth day, the difference becomes more pronounced as the leaves of Group 2 fully unfold. On the seventh day, the leaves of Group 2 are significantly larger and greener than those of Group 1. By the eighth day, differences in leaf color and seedling height become more apparent. As shown in Figure 6e,f, the weight and height of the corn seedlings in Group 2 consistently surpass those of Group 1. The prepared far‐red LEDs presents a positive effect in plant growth lighting, comparable to other successful lighting systems.^[^ ^26^, ^56^ ^]^ The results demonstrate that the prepared far‐red phosphor GSGO:60%Eu^3+^ can serve as effective light source for promoting plant growth.

## 3 Conclusion

In summary, two anomalously efficient far‐red phosphors A_3_Sc_2_C_3_O_12_ (A = Y^3+^, Gd^3+^. C = Al^3+^, Ga^3+^):Eu^3+^ were developed. The direct evidences from STEM‐HAADF confirm that the synergistic effect of distorted Eu^3+^ square antiprism occupancy and high concentration induces efficient far‐red emission. Concurrently, both materials exhibit extremely high thermal quenching resistance, which is attributed to high structural rigidity according to the theoretical calculations. The substitution of Gd^3+^‐Ga^3+^ for Y^3+^‐Al^3+^ induces the reduction of [EuO_8_] symmetry, acquiring the effective modulation of ^5^D_0_→^7^F_4_/^7^F_2_ transitions. The high quantum efficiency (IQE = 99.01% and EQE = 38.73%) of GSGO:60%Eu^3+^ is realized. The GSGO:60%Eu^3+^‐based far‐red LEDs exhibits excellent far‐red output power of 113 mW at 300 mA. The growth records of corn seeds and seedlings confirm the positive impact of the fabricated far‐red LEDs on plant growth lighting. This discovery paves the way for the design of highly efficient far‐red luminescence of Eu^3+^.

## 4 Experimental Section

### Synthesis

All the powder samples were synthesized using a high‐temperature solid‐state method. Y_2_O_3_ (99.99%), Gd_2_O_3_ (99.99%), Sc_2_O_3_ (99.99%), Al_2_O_3_ (99%), Ga_2_O_3_ (99.99%), and Eu_2_O_3_ (99.99%) were employed as starting materials. To facilitate the reaction, 3 wt% H_3_BO_3_ (99%) was added as flux. These reagents were precisely weighed according to their stoichiometric ratios and thoroughly ground for 60 min. The mixture was sintered at 1500 °C for 15 h. Finally, the sintered product was reground to yield the final white powder.

### Characterization

The phase identification was performed using a D8 ADVANCE XRD diffractometer (Cu Kα, λ = 0.15406 nm). The test was performed at a scan speed of 2°min^−1^ within the range of 10–90°. GSAS software was employed for the XRD Rietveld refinement analysis. The atomic‐structure information was observed by a high‐resolution STEM equipped with an aberration corrector in the condenser lens system (Thermofisher FEI Spectra 300). The morphological features were characterized using a Zeiss Gemini 500 SEM. The elemental composition was determined using EDS attached to the SEM. DR spectra were measured using a UV‐visible diffuse reflectance spectrometer (SHIMADZU UV2600). The photoluminescence properties (PLE, PL, decay time, and QE) were recorded on a fluorescence spectrometer (Edinburgh FS5) equipped with a xenon lamp. Temperature‐dependent emission spectra were measured through a HITACHI F‐7100 fluorescence spectrometer with a temperature controller. The optoelectronic properties of the resulting far‐red LEDs were tested using a HAAS2000 LED spectroscopy tester. The working temperatures of the far‐red LEDs were recorded by a forward‐looking infrared camera (FLIR Systems, AB).

### Fabrication of Far‐Red LEDs

The far‐red phosphor GSGO:60%Eu^3+^ and epoxy resin were accurately weighed in a 1:1 ratio and thoroughly stirred. The resulting mixture was coated on a NUV chip (395 nm) and dried in an oven at 80 °C for 2 h to obtain the far‐red LEDs.

### Experimental Process of Corn Growth

(i) The corn seeds were soaked in clean water for 24 h to ensure full hydration, creating an optimal foundation for subsequent growth. (ii) Then, the seeds were evenly divided into two groups of equal weight (16 seeds per group). (iii) Group 1 (control group) was placed under natural light, while Group 2 (experimental group) was placed under natural light supplemented with light from the prepared far‐red LEDs. To accurately evaluate the effects of different light sources on corn growth, both Groups maintained the identical light intensity (100 µmol m^−2^ s^−1^), light duration (8 a.m. to 8 p.m.), and environmental conditions. Light supplement method: Use an experimental stand with a length of 100 cm, a width of 55 cm, and a height of 70 cm, equipped with the prepared far‐red LEDs (voltage: 220 V, power: 20 W) at the top.

### Computational Methods

All DFT calculations were carried out using the Vienna ab‐initio simulation package (VASP). The electron‐ion interaction was described by the projector augmented wave (PAW) potentials. The exchange‐correlation functionals were represented by the generalized gradient approximation (GGA) with Perdew‐Burke‐Ernzerhof (PBE) scheme. A cut‐off energy of 500 eV was set to expand the electron wavefunctions. A 3 × 3 × 3 Monkhorst–Pack k‐points grid was uesd to sample the Brillouin‐zone. The convergence criteria were set as 0.02 eV Å^−1^ and 10^−6^ eV for structure relaxation and electronic energy calculaition, respectively.

### Statistical Analysis

Measurements of optical and plant growth properties, including IQE, EQE, and AE, as well as the weight and height of corn, were repeated at least three times to ensure the stable results. Standard deviations were included to account for measurement uncertainties.

## Conflict of Interest

The authors declare no conflict of interest.

## Supporting information



Supporting Information

## Data Availability

The data that support the findings of this study are available on request from the corresponding author. The data are not publicly available due to privacy or ethical restrictions.
