# Supplementary material for: Synergetic Contributions of High Quenching Concentration and Tuned Square Antiprism Geometry Boosting Far‐Red Emission of Eu3+ with Near‐Unit Efficiency
Source: Adv Sci (Weinh). 2025 Jan 10;12(9):2415989. doi: 10.1002/advs.202415989 (PMC11884608; doi:10.1002/advs.202415989)
Supplement: Supplementary file 1 — Supporting Information [file ADVS-12-2415989-s001.pdf]

ADVANCED SCIENCE
Open Access

Supporting Information

for *Adv. Sci.*, DOI 10.1002/advs.202415989

Synergetic Contributions of High Quenching Concentration and Tuned Square Antiprism Geometry Boosting Far-Red Emission of Eu^{3+} with Near-Unit Efficiency

Hong Li, Asif Ali Haider, Zhi Xie, Conglin Liu, Hongzhi Zhang*, Hongming Jiang, Junpeng Li and Jing Zhu**

Supporting Information

Synergetic Contributions of High Quenching Concentration and Tuned Square Antiprism Geometry Boosting Far-Red Emission of Eu^{3+} with Near-Unit Efficiency

Hong Li, Asif Ali Haider, Zhi Xie*, Conglin Liu, Hongzhi Zhang*, Hongming Jiang, Junpeng Li, Jing Zhu*

Dr. H. Li, A. A. Haider, C. L. Liu, H. Z. Zhang, H. M. Jiang, J. P. Li, Prof. J. Zhu
Yunnan Key Laboratory of Electromagnetic Materials and Devices, National Center for International Research on Photoelectric and Energy Materials, School of Materials and Energy, Yunnan University, Kunming 650091, China
E-mail: zhanghz@ynu.edu.cn; jzhu@ynu.edu.cn

Dr. Z. Xie
College of Mechanical and Electrical Engineering, Fujian Agriculture and Forestry University Fuzhou 350002,China
E-mail: xz@fafu.edu.cn

1. Supporting Figures

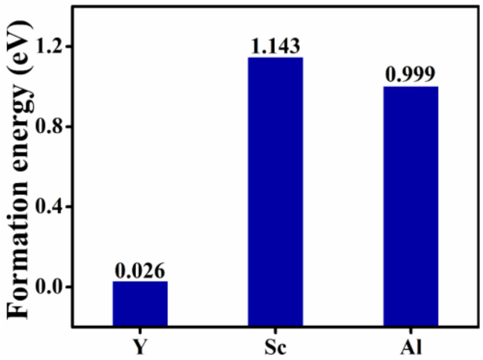

Figure S1. Formation energies of Eu^{3+} occupying Y^{3+}, Sc^{3+}, and Al^{3+} sites.

The X-ray diffraction (XRD) patterns of YSAO:$x$$Eu^{3+}$ (x = 0–100 mol%) and the standard one (JCPDS 79-1846 for YSAO) keep consistent (Figure S2a). No impurity peaks are observed, indicating that all samples maintain the crystal structure of YSAO. The XRD data were refined using General Structure Analysis System (GSAS) software according to the YSAO structure. The refined XRD pattern of YSAO is shown in Figure S2b. The structural parameters and refined results are listed in Table S1, confirming acceptable phase purity. The detailed bond lengths obtained by the Rietveld refinement are listed in Table S2. The fractional atomic coordinates and isotropic atomic displacement parameters are presented in Table S3.

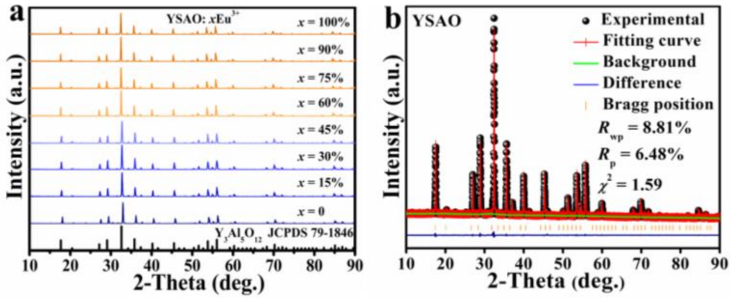

Figure S2. a) XRD patterns of YSAO:$x$$Eu^{3+}$ (x = 0–100 mol%). b) Rietveld refinement pattern of YSAO.

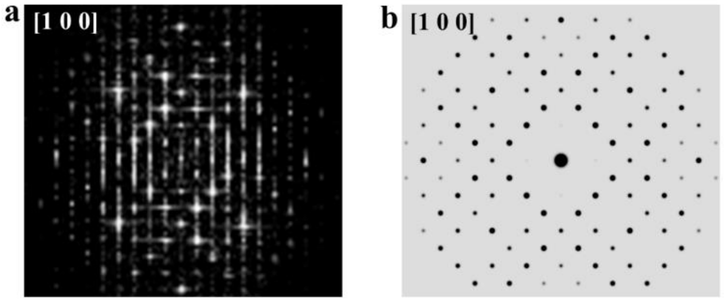

Figure S3. a) Electron diffraction pattern along the [1 0 0] zone axis. b) Simulated electron diffraction pattern along the [1 0 0] zone axis.

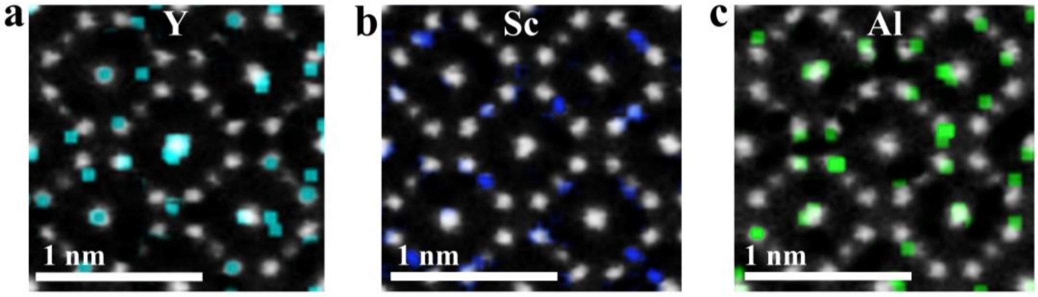

Figure S4. EDS mappings of a) Y^{3+}, b) Sc^{3+}, and c) Al^{3+} at atomic scale.

The EDS spectrum of YSAO:60%Eu^{3+} is shown in Figure S5a. All elements are detected without any impurity elements. Due to the use of a Si wafer as the substrate, a strong signal for Si element appears at 1.74 KeV. The insets show scanning electron microscopy (SEM) images of YSAO:60%Eu^{3+} at scales of 100 and 20 μm, respectively. The sample consists of irregular particles with an approximate diameter of 4.73 μm. Elemental mappings indicate that all the elements are uniformly distributed throughout the particles (Figure S5b).

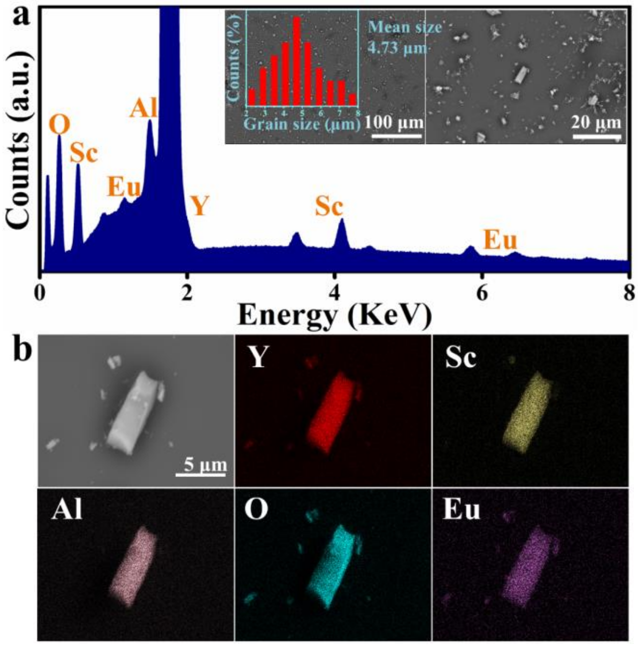

Figure S5. a) EDS pattern of YSAO:60%Eu^{3+} (Insets show the SEM images). b) Elemental mappings.

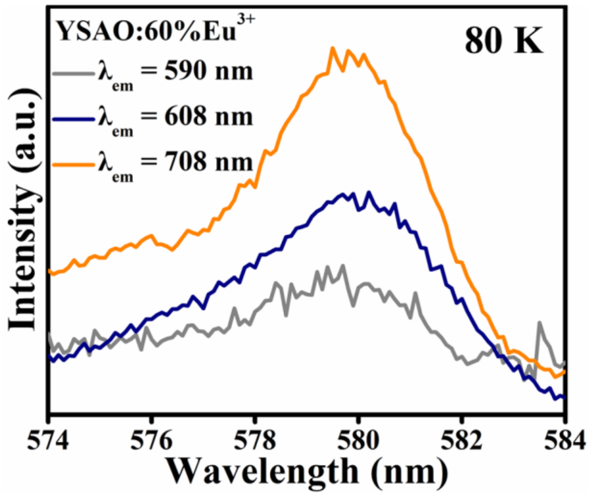

Figure S6. $^7F_0 \rightarrow {}^5D_0$ transition for YSAO:60%Eu^{3+} at 80 K.

The value of R_c is speculated based on the following formula proposed by Grabmaier and Blasse:[1]

$$R_c = 2(3V/4\pi\chi_c N)^{1/3} \quad \text{(S1)}$$

where V is the cell volume, χ_c denotes critical doping concentration, and N signifies the number of positions that the activator ions can occupy per unit cell. The exchange interaction dominates when R_c is less than 5 Å, otherwise the multipolar interaction prevails. In the case of YSAO:xEu^{3+}, V = 1880.57 Å^3, χ_c = 60 mol%, and N = 8. Thus, R_c is 9.08 Å. Therefore, the concentration quenching is attributed to electric multipolar interaction. The type of multipolar interaction can be determined using the Van Uitert model with the following equation:[2]

$$I/\chi = K[1 + \beta(\chi)^{\theta/3}]^{-1} \quad \text{(S2)}$$

where β and K are constants, I is emission intensity, and χ is Eu^{3+} doping concentration. θ can take the values of 6, 8, and 10, which represent the electric dipole-dipole, dipole-quadrupole, and quadrupole-quadrupole interactions, respectively. Because the value of $\beta(\chi)^{\theta/3}$ is significantly greater than 1, Formula S2 can be simplified as Formula S3:

$$\log(I/\chi) = A - (\theta/3)\log(\chi) \quad \text{(S3)}$$

where A is a constant, and θ value is determined via a linear relationship between $\log(I/\chi)$ and $\log(\chi)$. Figure S7 shows a plot based on Formula S2 and S3. The fitting results (θ = 4.35) suggest that the concentration quenching mechanism is due to the electric dipole-dipole interaction.

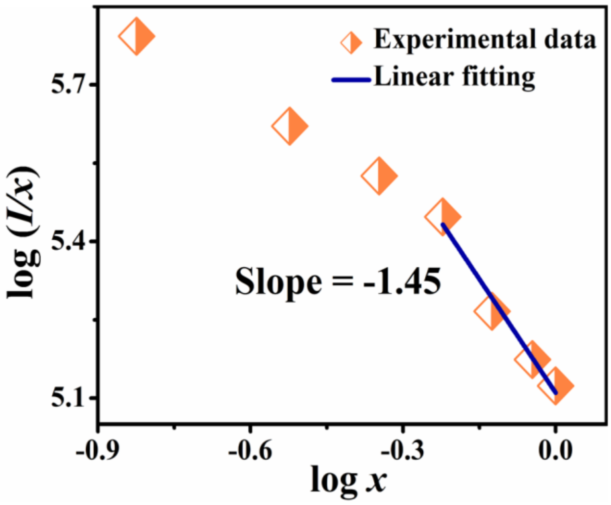

Figure S7. Variation of log(I/χ) with log(χ).

The initial quenching concentration can be obtained by 1/(1 + Z) according to Ozawa's theory[3], where Z is the number of closest cations around the luminescence centers. Additionally, there is a relationship between the emission intensity (I) and doping concentration of the luminescence center (χ):

$$1\text{n}\left(\frac{I}{\chi}\right) = Z1\text{n}(1-\chi) + C \qquad \text{(S4)}$$

Here, C is a constant. The value of Z can be obtained from the slope of ln(I/χ) ~ ln(1-χ). In YSAO:Eu^{3+}, the relationship between ln(I/χ) and ln(1-χ) is shown in Figure S8. Z is found to be 0.6293 by linear fitting. Therefore, the theoretical quenching concentration is 61.37%.

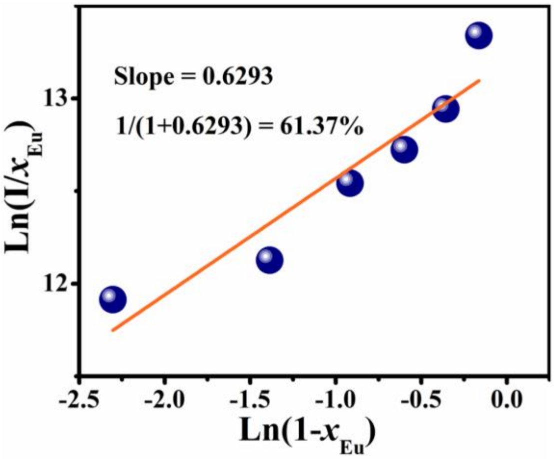

Figure S8. Relationship of ln(I/x_{Eu}) ~ ln(1-x_{Eu}).

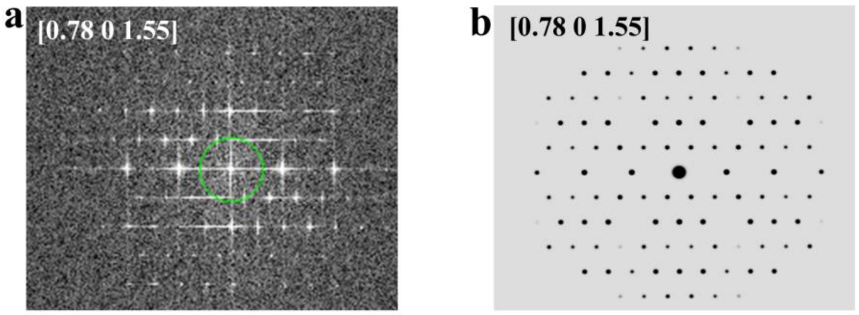

Figure S9. a) Electron diffraction pattern along the [0.78 0 1.55] zone axis. b) Simulated electron diffraction pattern along the [0.78 0 1.55] zone axis.

The XRD patterns of GSGO and GSGO:60%Eu^{3+} are shown in Figure S10a, indicating that both samples have the same structure with YSAO. The refined XRD patterns are shown in Figure S10b,c. The structural parameters and refined results are listed in Table S1, where the low *R* factors confirm the phase purity. The detailed bond lengths obtained by Rietveld refinement are shown in Table S2. The fractional atomic coordinates and isotropic atomic displacement parameters are shown in Table S3. The EDS pattern of GSGO:60%Eu^{3+} is shown in Figure S10d. All anticipated elements are detected with no impurity elements. The strong peak at 1.74 KeV is attributed to the Si substrate. The insets show the SEM images at scales of 100 and 20 μm. GSGO:60%Eu^{3+} consists of irregular particles with an approximate diameter of 6.93 μm. The elemental mappings show that all elements are uniformly distributed throughout the particles (Figure S10e). These results indicate that GSGO and GSGO:60%Eu^{3+} are successfully synthesized.

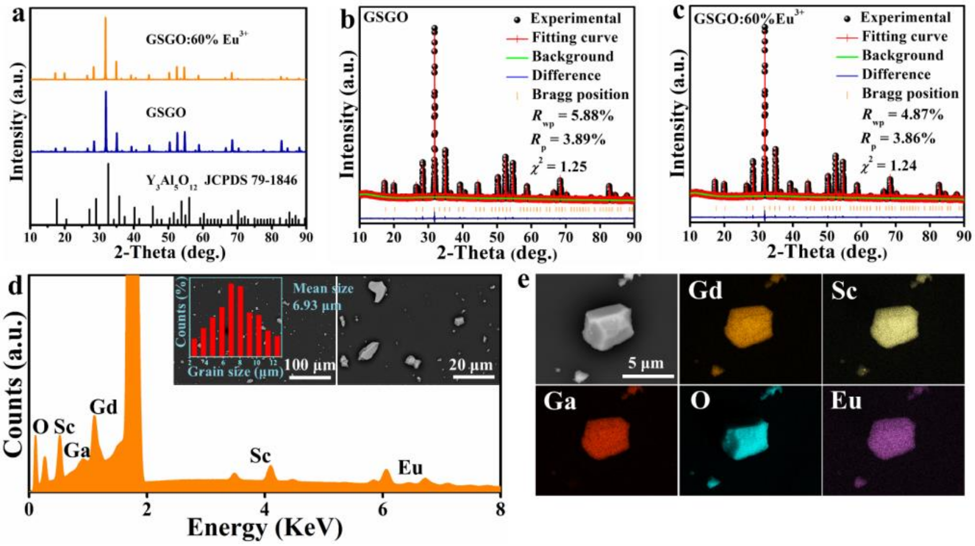

Figure S10. a) XRD patterns of GSGO and GSGO:60%Eu^{3+}. Rietveld refinement patterns for b) GSGO and c) GSGO:60%Eu^{3+}. d) EDS of GSGO:60%Eu^{3+} (Insets show the SEM images). e) Elemental mappings.

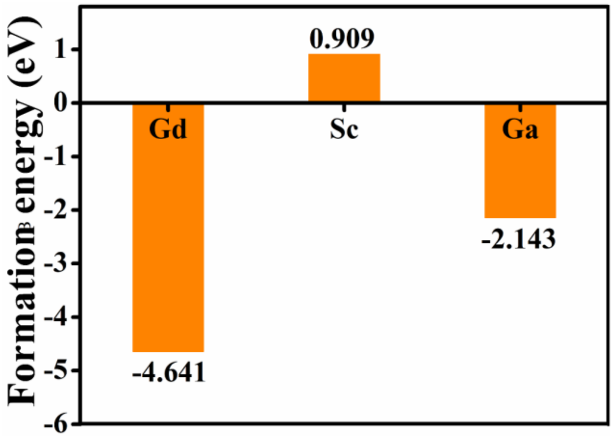

Figure S11. Formation energies of Eu^{3+} occupying the Gd^{3+}, Sc^{3+}, and Ga^{3+} sites.

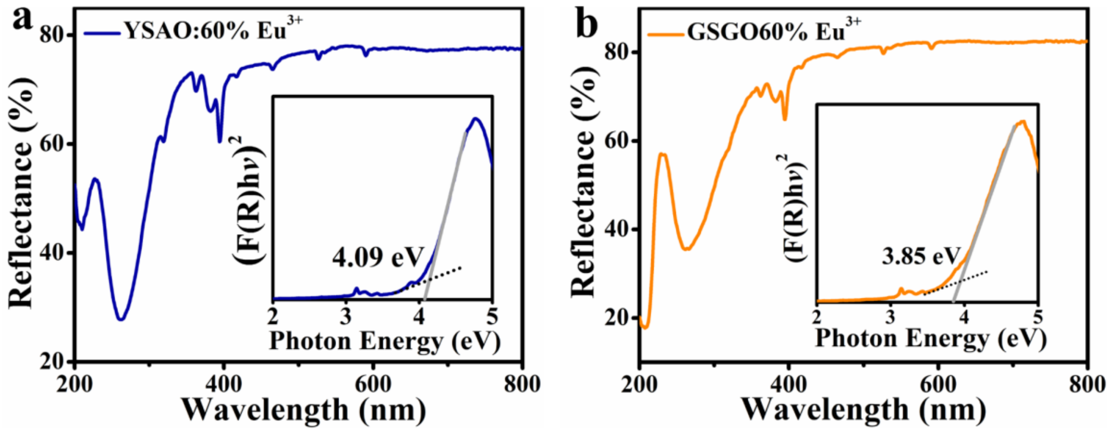

Figure S12. a) DR spectra of a) YSAO:60%Eu^{3+} and b) GSGO:60%Eu^{3+} (Insets show the experimental determinations of E_g).

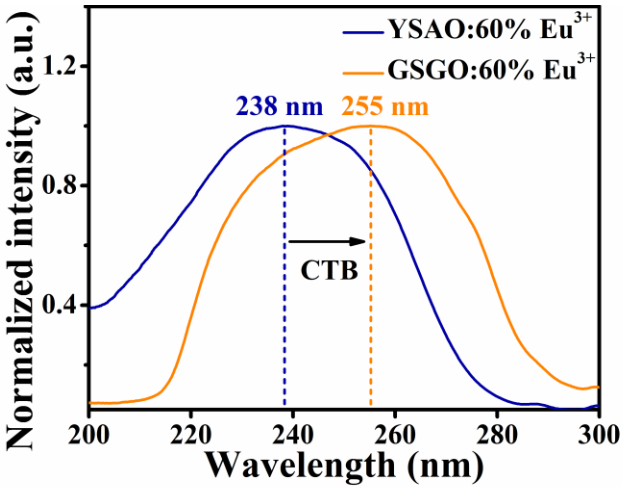

Figure S13. CTB comparison of YSAO:60%Eu^{3+} and GSGO:60%Eu^{3+}.

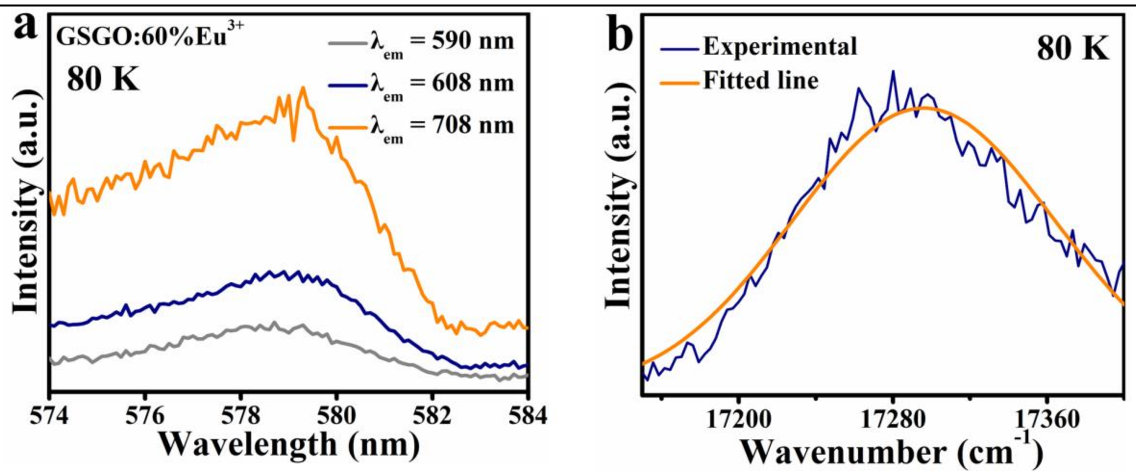

Figure S14. a) $^7F_0 \rightarrow {}^5D_0$ transition for GSGO:60%Eu^{3+} at 80 K. b) Gaussian fitting diagram (λ_{em} = 708 nm).

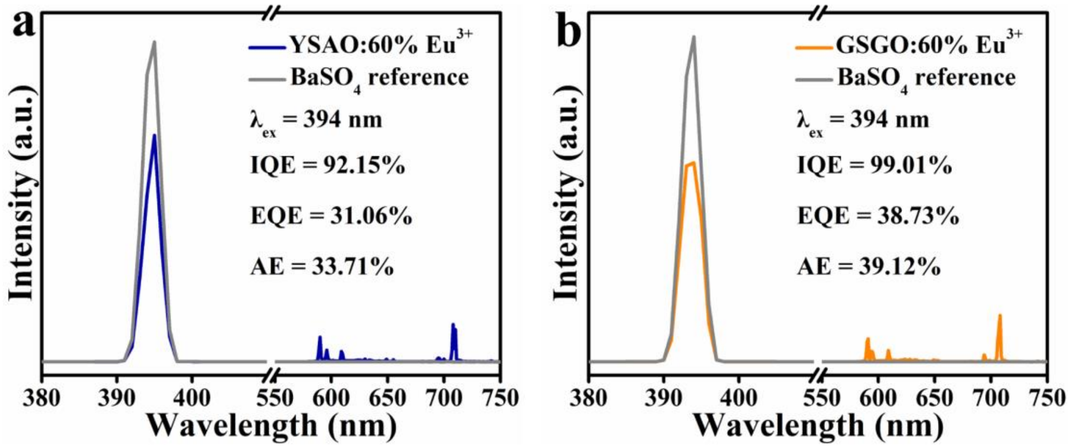

Figure S15. QE test spectra of a) YSAO:60%Eu^{3+} and b) GSGO:60%Eu^{3+}.

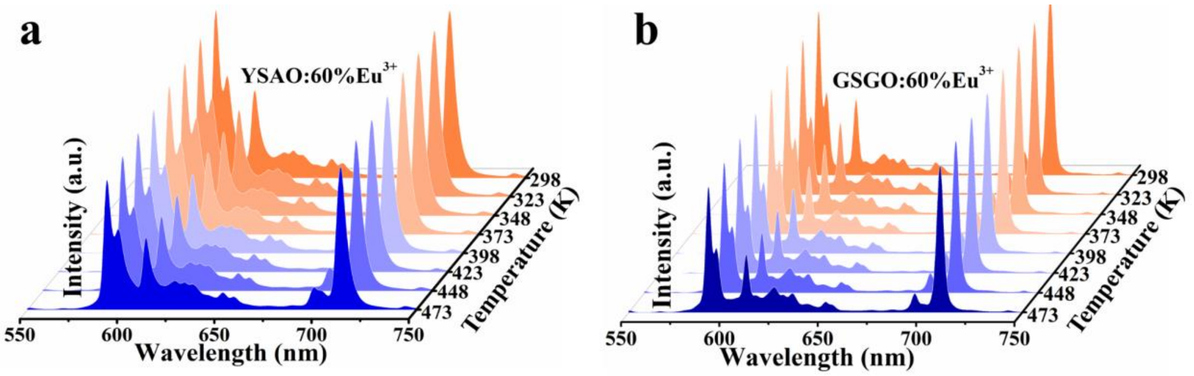

Figure S16. Temperature-dependent PL spectra of a) YSAO:60%Eu^{3+} and b) GSGO:60%Eu^{3+}.

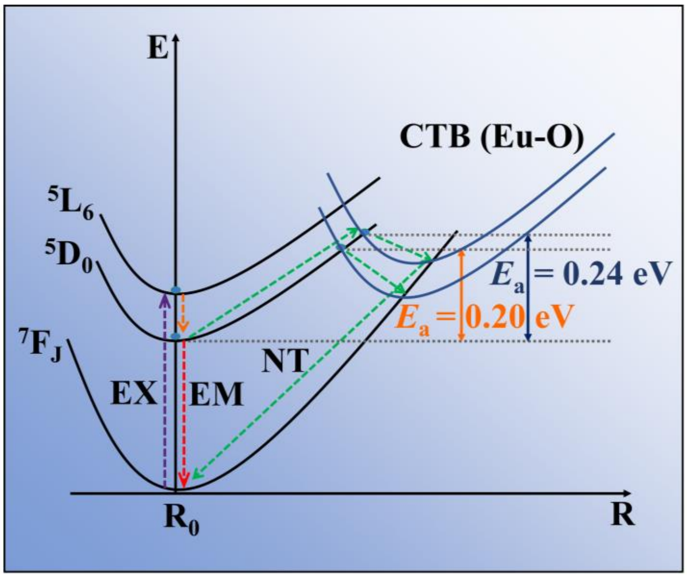

Figure S17. Configurational coordinate diagram.

2. Supporting Tables

Table S1. XRD Rietveld refinements and lattice parameters of YSAO, YSAO:60%Eu^{3+}, GSGO, and GSGO:60%Eu^{3+}.

	YSAO	YSAO:60%Eu^{3+}	GSGO	GSGO:60%Eu^{3+}
**Cell Parameters**				
a = b = c (Å)	12.3253	12.3433	12.5558	12.5672
α = β = γ	90°	90°	90°	90°
V (Å^3)	1872.383	1880.570	1979.403	1984.776
Crystal system	Cubic			
Space group	$Ia\bar{3}d$ (230)			
**Covariance**				
R_{wp}%	8.81	6.42	5.88	4.87
R_p%	6.48	4.89	3.89	3.86
χ^2	1.59	1.54	1.25	1.24

Table S2. The bond lengths of YSAO, YSAO:60%Eu^{3+}, GSGO, and GSGO:60%Eu^{3+} obtained through the Rietveld refinement.

	**YSAO**	**YSAO:60%Eu^{3+}**
Y1-O1	2.432(3)	2.444(3)
Y1-O1	2.371(3)	2.477(3)
Y1-O1	2.432(3)	2.444(3)
Y1-O1	2.432(3)	2.477(3)
Y1-O1	2.371(3)	2.477(3)
Y1-O1	2.371(3)	2.444(3)
Y1-O1	2.371(3)	2.477(3)
Y1-O1	2.432(3)	2.444(3)
Average bond length	2.4017	2.4604
	**GSGO**	**GSGO:60%Eu^{3+}**
Gd1-O1	2.514(4)	2.525(5)
Gd1-O1	2.489(3)	2.525(5)
Gd1-O1	2.514(4)	2.604(5)
Gd1-O1	2.489(3)	2.604(5)
Gd1-O1	2.489(3)	2.604(5)
Gd1-O1	2.514(4)	2.604(5)
Gd1-O1	2.489(3)	2.525(5)
Gd1-O1	2.514(4)	2.525(5)
Average bond length	2.5013	2.5647

Table S3. Fractional atomic coordinates, occupancy factors, and isotropic displacement parameters (Å^2) of YSAO, YSAO:60%Eu^{3+}, GSGO, and GSGO:60%Eu^{3+}.

Atom	Wyckoff position	*x*	*y*	*z*	Occ.	U_{iso}
			**YSAO**			
Y	24c	0	0.25	0.125	1	0.05477(28)
Sc	16a	0	0	0	1	0.0534(8)
Al	24d	0	0.25	0.375	1	0.0566(9)
O	96h	0.0334(3)	0.0582(8)	0.6577(4)	1	0.0544(11)
			**YSAO:60%Eu^{3+}**			
Y	24c	0	0.25	0.125	0.8	0.00658(23)
Sc	16a	0	0	0	1	0.0028(7)
Al	24d	0	0.25	0.375	1	0.0079(8)
O	96h	0.0425(1)	0.0592(4)	0.6567(5)	1	0.0066(12)
Eu	24c	0	0.25	0.125	0.2	0.00658(23)
			**GSGO**			
Gd	24c	0	0.25	0.125	1	0.0087(7)
Sc	16a	0	0	0	1	0.02162(21)
Ga	24d	0	0.25	0.375	1	0.0179(4)
O	96h	0.0393(8)	0.0559(0)	0.6544(0)	1	0.0117(13)
			**GSGO:60%Eu^{3+}**			
Gd	24c	0	0.25	0.125	0.8	0.02056(28)
Sc	16a	0	0	0	1	0.0060(9)
Ga	24d	0	0.25	0.375	1	0.0160(5)
O	96h	0.0425(2)	0.0488(4)	0.6503(4)	1	0.0291(19)
Eu	24c	0	0.25	0.125	0.2	0.02056(28)

Table S4. Comparison of luminescence properties of reported Eu^{3+}-activated garnet phosphors with those of YSAO:60%Eu^{3+} and GSGO:60%Eu^{3+}.

Phosphors	λ_{em} (nm)	IQE (%)	EQE (%)	AE (%)	$I_{150℃}$ (%)	Ref.
$Y_3Al_5O_{12}:Eu^{3+}$	708	23.73	-	-	88.9	[4]
$Y_2Mg_2Al_2Si_2O_{12}:Eu^{3+}$	707	60.5	-	-	88.5	[4]
$Ca_2LaHf_2Al_3O_{12}:Eu^{3+}$	614	64	-	-	73.7	[5]
$Ca_3Zr_2SiGa_2O_{12}:Eu^{3+}$	610	39.65	-	-	96.5	[6]
$Ca_2GdHf_2Al_3O_{12}: Eu^{3+}$	616	53.5	-	-	79	[7]
$Ca_{3.5}LuHf_{0.5}Ge_3O_{12}:Eu^{3+}$	610	91.2	-	-	83.9	[8]
$NaGd_2Ga_3Ge_2O_{12}:Eu^{3+}$	707	96.78	-	-	82.87	[9]
$CaY_2Al_4SiO_{12}:Eu^{3+}$	592	17.5	-	-	90	[10]
$(Gd_{0.85}Lu_{0.1}Eu_{0.05})_3Al_5O_{12}$	710	42	-	-	76.1	[11]
$Ca_2LuZr_2Al_3O_{12}:Eu^{3+}$	618	/	/	/	86	[12]
$NaY_2Ga_2InGe_2O_{12}:Eu^{3+}$	707	95.6	/	/	79.4	[13]
$Ca_2LuHf_2Al_3O_{12}:Eu^{3+}$	611	39.2	/	/	71	[14]
$Ca_2YHf_2Al_3O_{12}:Eu^{3+}$	611	56.6	/	/	75	[14]
$Ca_3Al_2Ge_3O_{12}:Eu^{3+}$	707	98.4	/	/	78.8	[15]
$Y_2SrAl_4SiO_{12}:Eu^{3+}$	710	/	/	/	92	[16]
$Ca_2KMg_2V_3O_{12}:Eu^{3+}$	612	62.8	/	/	75.4	[17]
$Na_{2.5}Zr_2Si_{1.5}P_{1.5}O_{12}$:Eu^{3+}	613	72	15	21	93.3	[18]
**$Y_3Sc_2Al_3O_{12}:Eu^{3+}$**	**708**	**92.15**	**31.06**	**33.71**	**96**	**Tw**
**$Gd_3Sc_2Ga_3O_{12}:Eu^{3+}$**	**708**	**99.01**	**38.73**	**39.12**	**90**	**Tw**

Table S5. Comparison of output power and photoelectric conversion efficiency of the far-red LED devices.

Far-red phosphors	λ_{em} (nm)	NIR output power (mW) @300 mA	photoelectric efficiency (%) @300 mA	Ref.
$Mg_{1.4}Zn_{0.6}Sn_{0.7}Ti_{0.3}O_4:Cr^{3+}$	752	900	2.6	[19]
$Ca_2YAl_3Ge_2O_{12}:Cr^{3+}$	770	80	8.3	[20]
$BaY_2Ga_{3.9}GeO_{12}:Cr^{3+}$	710	62	6.5	[21]
$K_3AlF_6:Cr^{3+}$	750	7-8	<1	[22]
$La_2MgTiO_6:Cr^{3+}$	762	8.3	/	[23]
$La_2MgTiO_6:Mn^{4+}$	710	6.1	/	[23]
$MgAl_2O_4:Mn^{4+}$	825	78.41@120 mA	2.42@120 mA	[24]
$K_3LuSi_2O_7:Eu^{2+}$	740	21.5	7.1	[25]
$Ca_3Al_2Ge_3O_{12}:Eu^{3+}$	707	27.3@200 mA	4.0@200 mA	[15]
$GdTeBO_5:Eu^{3+}$	699	120	4.9	[26]
**$Gd_3Sc_2Ga_3O_{12}:Eu^{3+}$**	**708**	**113**	**4.37**	**Tw**

3. References

[1] G. Blasse, *Phys. Lett. A* **1968**, *28*, 444–445.

[2] Q. Wang, S. W. Wang, T. Tan, J. T. Wang, R. Pang, D. Li, C. Y. Li, H. J. Zhang, *Inorg. Chem. Front.* **2022**, *9*, 3692–3701.

[3] L. Ozawa, *J. Electrochem. Soc.* **1979**, *126*, 106–109.

[4] X. Zhang, T. Shen, D. Kan, D. Zhang, R. Dong, Z. An, Y. Song, K. Zheng, Y. Sheng, Z. Shi, H. Zou, *Inorg. Chem.* **2020**, *59*, 9927−9937.

[5] N. Ma, W. Li, B. Devakumar, X. Huang, *Inorg. Chem.* **2022**, *61*, 6898−6909.

[6] S. He, S. Ma, Q. Guan, S. Zheng, L. Chao, J. Qiao , Y. Ma, *J. Lumin.* **2024**, *269*, 120454.

[7] Y. Li, Q. Wei, H. Chen, Y. Wang, *J. Alloy. Compd.* **2021**, *864*, 158840.

[8] Y. Wang, X. Chen, K. Ma, W. Geng, X. Zhou, T. Liu, S. Xu, X. Zhang, Y. Cao, J. Zhang, B. Chen, *Ceram. Int.* **2024**, *50*, 6648–6657.

[9] F. Xie, J. Gu, S. Zhong, P. Zhang, Y. Wen, Y. Li, H. Xu, S. Yao, Q. Zhang, J. Li, *Mater. Today Chem.* **2024**, *37*, 102019.

[10] A. Verma, R. Sharma, D. Bisen, N. Brahme, T. Richhariya, K. Tiwari, K. Thakkar, *J. Electron. Mater*. **2023**, *52*, 6769–6777.

[11] Z. Sun, J. Li, H. Qian, Y. Sakka, T. Suzuki, B. Lu, *J. Adv. Ceram.* **2024**, *13*, 113−123.

[12] X. Huang, L. Cao, B. Devakumar, *Inorg. Chem. Commun*. **2024**, *162*, 112232.

[13] J. Feng, L. Chen, J. Xie, Q. Zhang, Y. Yu, L. Luo, Q. Tang, J. Zhou, J. Li, *Mater. Today Chem.* **2024**, *36*, 101979.

[14] N. Ma , W. Li, X. Huang, *J. Lumin.* **2024**, *265*, 120232.

[15] S. Miao, R. Shi, Y. Zhang, D. Chen, Y. Liang, *Adv. Mater. Technol.* **2023**, *8*, 2202103.

[16] X. Chen, Q. Xu, F. Hussain, C. Yang, W. Sheng, X. Luo, B. Liu, S. Sun, D. Wang, K. Song, *Crystals* **2022**, *12*, 1382.

[17] A. Princy, V. Mala, K. Albert, S. Kennedy, *Inorg. Chem. Commun.* **2023**, *158*, 111574.

[18] B. Shao, Q. Lv, X. Ma, Y. Li, X. Zhou, C. Wang, Y. Wang, *J. Lumin.* **2022**, *247*, 118912.

[19] S. Gai, C. Zhou, L. Peng, M. Wu, P. Gao, L. Su, M. Molokeev, Z. Zhou, M. Xia, *Mater. Today Chem.* **2022**, *26*, 101107.

[20] X. Dai, X. Zou, H. Zhang, W. Chen, C. Yang, M. Molokeev, Z. Xia, Y. Liu, X. Zhang, M. Zheng, B. Lei, *Adv. Opt. Mater.* **2024**, *12*, 2302380.

[21] X. Dai, X. Zou, M. Wei, X. Zhang, B. Dong, X. Li, Y. Cong, D. Li, J. Zhao, M. Molokeev, B. Lei, *Adv. Opt. Mater.* **2024**, *12*, 2401608.

[22] C. Lee, Z. Bao, M. Fang, T. Lesniewski, S. Mahlik, M. Grinberg, G. Leniec, S. Kaczmarek, M. Brik, Y. Tsai, T. Tsai, R. Liu, *Inorg. Chem.* **2020**, *59*, 376−385.

[23] D. Zheng, X. Li, J. Chen, J. Zhang, K. Gao, Y. Zhu, D. Hou, R. Huang, J. Li, J. Dong, *J. Lumin.* **2023**, *257*, 119778.

[24] E. Song, X. Jiang, Y. Zhou, Z. Lin, S. Ye, Z. Xia, Q. Zhang, *Adv. Opt. Mater.* **2019**, *7*, 1901105.

[25] J. Qiao, G. Zhou, Y. Zhou, Q. Zhang, Z. Xia, *Nat. Commun.* **2019**, *10*, 5267.

[26] H. Li, Y. Niu, C. Liu, H. Jiang, J. Li, J. Wu, S. Huang, H. Zhang, J. Zhu, *Laser Photonics Rev.* **2024**, *18*, 2400843.
